# Toll-like receptor expression in pulmonary sensory neurons in the bleomycin-induced fibrosis model

**DOI:** 10.1371/journal.pone.0193117

**Published:** 2018-03-08

**Authors:** Won Jai Jung, Sang Yeub Lee, Sue In Choi, Byung-Keun Kim, Eun Joo Lee, Kwang Ho In, Min-Goo Lee

**Affiliations:** 1 Division of Pulmonology, Allergy and Critical Care Medicine, Department of Internal Medicine, Korea University College of Medicine, Seoul, Korea; 2 Department of Physiology, Korea University College of Medicine, Seoul, Korea; West Virginia University School of Medicine, UNITED STATES

## Abstract

Airway sensory nerves are known to express several receptors and channels that are activated by exogenous and endogenous mediators that cause coughing. Toll-like receptor (TLR) s are expressed in nociceptive neurons and play an important role in neuroinflammation. However, there have been very few studies of TLR expression in lung-derived sensory neurons or their relevance to respiratory symptoms such as cough. We used the bleomycin-induced pulmonary fibrosis model to investigate the change in TLR expression in pulmonary neurons and the association of TLRs with transient receptor potential (TRP) channels in pulmonary neurons. After 2 weeks of bleomycin or saline administration, pulmonary fibrosis changes were confirmed using tissue staining and the SIRCOL collagen assay. TLRs (TLR 1–9) and TRP channel expression was analyzed using single cell reverse transcription polymerase chain reaction (RT-PCR) in isolated sensory neurons from the nodose/jugular ganglion and the dorsal root ganglion (DRG). Pulmonary sensory neurons expressed TLR2 and TLR5. In the bleomycin-induced pulmonary fibrosis model, TLR2 expression was detected in 29.5% (18/61) and 26.9% (21/78) of pulmonary nodose/jugular neurons and DRG neurons, respectively. TLR5 was also detected in 55.7% (34/61) and 42.3% (33/78) of pulmonary nodose/jugular neurons and DRG neurons, respectively, in the bleomycin-induced pulmonary fibrosis model. TLR5 was expressed in 63.4% of TRPV1 positive cells and 43.4% of TRPM8 positive cells. In conclusion, TLR2 and TLR5 expression is enhanced, especially in vagal neurons, in the bleomycin-induced fibrosis model group when compared to the saline treated control group. Co-expression of TLR5 and TRP channels in pulmonary sensory neurons was also observed. This work sheds new light on the role of TLRs in the control and manifestation of clinical symptoms, such as cough. To understand the role of TLRs in pulmonary sensory nerves, further study will be required.

## Introduction

The sensory nerves that innervate the respiratory tract are known to express several receptors and channels that are activated by exogenous and endogenous mediators in respiratory disease. Activation of certain vagal afferent nerves in the respiratory tract can lead to the conscious sensations of dyspnea and the urge to cough [1]. The ion channels like transient receptor potential (TRP) channels on vagal sensory afferents are involved in initiating cough [2]. Recent literature has reported that the expression of some TRP channels is increased in lung disease and that this increase is associated with the development of cough [3, 4].

The toll-like receptor (TLR) is a pattern-recognition receptor that not only plays a major role in regulating immune responses but also responds to the products of tissue damage, thereby playing a major role in inflammation [5–7]. Additionally, TLRs are expressed in nociceptive neurons and play an important role in neuroinflammation. The expression and function of several TLRs in neuronal cells has been demonstrated recently [8–10]. Some experts proposed that cough, pain and itch had shared mechanisms of neuro-immune interaction. In these different conditions, ion channels such as TRPA1 and Nav1.8, and TLRs were implicated in peripheral sensitization and neurogenic inflammation [11]. However, investigation of TLRs in pulmonary sensory neurons or the association between TLRs, TRP channels and respiratory symptoms, such as cough, have not been studied sufficiently.

Pulmonary fibrosis is a component of diverse interstitial lung disease (ILD), including idiopathic pulmonary fibrosis (IPF), which is a chronic, and progressive disease. This disease has a poor prognosis, with a mean survival period of 2–4 years and there is currently no effective therapy. This disease is characterized by refractory respiratory symptoms including mainly cough and dyspnea; more than 80% of patients with idiopathic pulmonary fibrosis suffer from cough [12]. Intractable cough and shortness of breath has a substantial negative impact on the quality of life. Bleomycin, one of the clinically relevant causative agents of pulmonary fibrosis, induces DNA strand breaks, resulting in pulmonary inflammation, injury, and subsequent interstitial fibrosis. Murine models of bleomycin-induced lung injury have been developed and used experimentally to understand the pathophysiology of pulmonary fibrosis [13].

To identify the expression and role of TLRs in pulmonary sensory neurons, we investigated the expression of TLRs and TRP channels in isolated rat pulmonary sensory neurons using single cell reverse transcription polymerase chain reaction (RT-PCR) in the bleomycin-induced fibrosis murine model. Additionally, we examined the expression of TRP channels and the co-expression of these channels and TLRs in the bleomycin-induced lung fibrosis model.

## Materials and methods

### Animals

Pathogen-free Sprague-Dawley rats (Samtako, Korea) weighing 200–250 g were used at 7 weeks of age and housed in the laboratory animal facility of the medical college of Korea University. Rats were anesthetized for the experiment with 50 mg/kg of ketamine and 20 mg/kg of xylazine. All experimental protocols were approved by the Institutional Animal Care and Use Committee of Korea University.

### Bleomycin-induced lung fibrosis model

The bleomycin-rat model of lung fibrosis was used [14–16]. Rats (bleomycin group, *n* = 10; control group, *n* = 3) were injected with a single dose of bleomycin (2.5 mg/kg body weight in sterile saline; bleomycin, Enzo life sciences, USA) or the same volume of saline via intratracheal instillation [16]. The rats were rotated immediately after bleomycin instillation to ensure thorough drug distribution in the lungs. After recovery from the anesthesia, the rats were returned to their cages and allowed food and water as normal. Fourteen days following intratracheal instillation, the animals were sacrificed using 100% CO_2_ asphyxiation, and the lung tissue was harvested for evaluation.

### Lung histological analysis and collagen assay

The left lung tissue was fixed, using 4% paraformaldehyde in PBS, and embedded in paraffin before being cut into 2-μm-thick sections. For histological examination, sections were stained with hematoxylin and eosin (H&E). Masson’s trichrome staining was performed to assess lung fibrosis. The histological changes were evaluated using a descriptive method [15].

The level of collagen in the lung tissue was determined using the SIRCOL collagen assay (Biocolor LTD., UK) according to manufacturer’s instructions. Briefly, right lung lobes were homogenized and collagen was solubilized in 0.5 M acetic acid. Tissue extracts were incubated with Sirius red dye and the absorbance was determined at 540 nm using a spectrophotometer. The amount of collagen was expressed in μg/g of wet tissue [16, 17].

### Labeling pulmonary sensory neurons and isolation of ganglionic neurons

Sensory neurons innervating the lungs and airways were identified using retrograde labeling from the lungs by the fluorescent tracer, 1,1′-dioctadecyl-3,3,3′,3′-tetramethylindocarbocyanine perchlorate (DiI) [18]. DiI (Vybrant Multicolor cell-labeling kit, Invitrogen, USA) solution (0.1%, dissolved in 1% of DMSO and 99% normal saline) was instilled into the trachea with bleomycin or normal saline.

Fourteen days after the intratracheal instillation, vagal (nodose, jugular) and dorsal root ganglia (DRG; Th7 to Th9) were extracted. Isolated ganglia were separately incubated in the enzyme buffers (2 mg/ml collagenase type 1A and 2 mg/ml dispase II in 2 ml Ca^2+^, Mg^2+^-free Hanks’ balanced salt solution) for 2 h at 37°C. Ganglionic neurons were dissociated by glass pipette trituration with three steps of decreasing tip pore size, then washed with culture media (DMEM containing 10% fetal bovine serum) by centrifugation (three times at 1500 g for 2 min) and suspended in 300 μl of the culture media. The dissociated neuron suspension was transferred onto 12 mm circular glass coverslips coated with poly D-lysine (20 μg/ml). After the neurons had adhered to the coverslips following 2 hours of incubation, the coverslips were flooded with the culture media and placed in a 5% CO_2_ incubator. The neuron-attached coverslips were used within 30 hours.

### Single cell RT-PCR

The neuron-attached coverslip was constantly superfused with Locke solution. A single neuron, which was identified for DiI labelling under the fluorescence microscope, was harvested into a glass pipette (tip diameter of 50–100 μm) by applying negative pressure. The pipette tip was immediately broken in a PCR tube containing resuspension buffer with RNase inhibitor and stored in liquid nitrogen. Five to ten labelled neurons were collected from one coverslip. Bath solution was also collected from the vicinity of a labelled neurons in each coverslip using the same method for a negative control. Later, the collected samples were defrosted, lysed (10 min at 75°C), treated with DNase I and reverse transcribed. Aliquots (2 μl) of the reaction product were used for PCR of Rat GAPDH (NCBI nucleotide *Rattus norvegicus* glyceraldehyde-3-phosphate dehydrogenase (Gapdh), mRNA searching forward primer: 572–590, TTGGCATCGTGGAAGGGCT.; reverse primer: 803–821 TTCTCCAGGCGGCATGTCA.; product length 249 bp), TLR2 (NCBI nucleotide *Rattus norvegicus* toll-like receptor 2 (Tlr2) mRNA, searching; forward primer: 34–54, TGGAGGTCTCCAGGTCAAATC.; reverse primer: 421–441, TTCAAAGAGGAAAGGGGCCTG.; product length 408 bp), TLR5 (NCBI nucleotide *Rattus norvegicus* toll-like receptor 5 (Tlr5) mRNA, searching forward primer: 3688–3708, TGTCTCGGTGACCTCCAAATG.; reverse primer: 4104–4123, GAGGTCCCGTGCAGAAGATG.; product length 436 bp) and TRPA1 (NCBI nucleotide *Rattus norvegicus* transient receptor potential cation channel, subfamily A, member 1 (Trpa1), mRNA searching; forward primer: 1682–1701, ATGCAAAGGCTGTTGCGATG.; reverse primer: 2100–2119, CCCTACACACAGGGTGGTTG.; product length 438 bp), TRPV1 (NCBI nucleotide *Rattus norvegicus* transient receptor potential cation channel, subfamily V, member 1 (Trpv1), mRNA, searching forward primer: 15–34, TTGCTCCATTTGGGGTGTGC.; reverse primer: 581–600, CGATGGTGTCATTCTGCCCA.; product length 586 bp), TRPM8 (NCBI nucleotide *Rattus norvegicus* transient receptor potential cation channel, subfamily M, member 8 (Trpm8), mRNA, searching; forward primer: 3411–3431, CAAACACCTGGATTTGGAGGC.; reverse primer: 3917–3937, TGGAGAGCCCCAGAG AAAGAA.; product length 527 bp) receptors. After the initial denaturation at 95°C for 2 min, the cDNA was amplified through 40 cycles of denaturation at 95°C for 20 s, annealing at 55°C for 40 s and extension at 72°C for 45 s followed by final elongation at 72°C for 5 min using *Taq* polymerase. The PCR products were visualized in ethidium bromide-stained 2% agarose gels [19–21].

### Statistical analysis

All results are presented as mean ± S.E.M. The Mann–Whitney test and χ^2^ test were performed using IBM SPSS Statistics 20.0 (IBM Corporation, NY, USA) and GraphPad Prism^®^ (GraphPad software, Inc., USA) for statistical analysis when appropriate. A *P*-value less than 0.05 was considered statistically significant.

## Results

### Histological analysis and collagen assay

**Fig 1:**
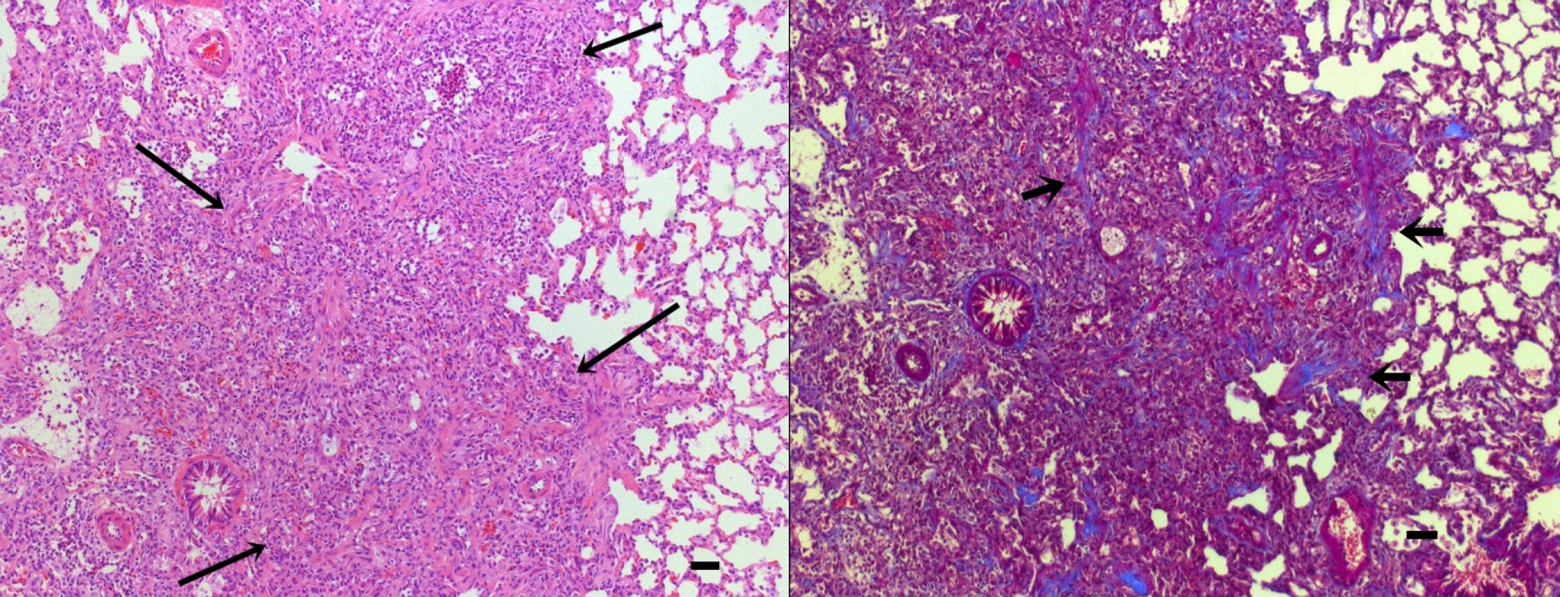
Representative images of lung tissue from rats in the bleomycin-treated group. Lung tissues were harvested 14d after instillation of bleomycin. On Left, hematoxylin and eosin (H&E) and on right masson trichrome–stained sections viewed at a magnification of ×100. Long Arrows indicate extensive infiltration of inflammatory cells and fibrosis. Short arrows indicate collagen stained blue. Bar = 100μm.

Hematoxylin-eosin and Masson’s trichrome stained lung sections were examined by light microscopy. Lung tissue from saline-treated control groups at 14 d showed little inflammatory reaction and scarce fibrosis. Conversely, at the same time point in the bleomycin-treated group infiltration of inflammatory cells, such as neutrophils and lymphocytes, was observed around the small vessels, terminal bronchioles, alveolar spaces, and alveolar septa. Collagen fibers were observed as a Masson trichrome stain deposited around the vessels, bronchioles, and alveolar septae where inflammatory changes occur. Several areas of widened collagen accumulation and intra-alveolar fibrosis were noted, distorting the normal parenchymal architecture (Fig 1).

**Fig 2:**
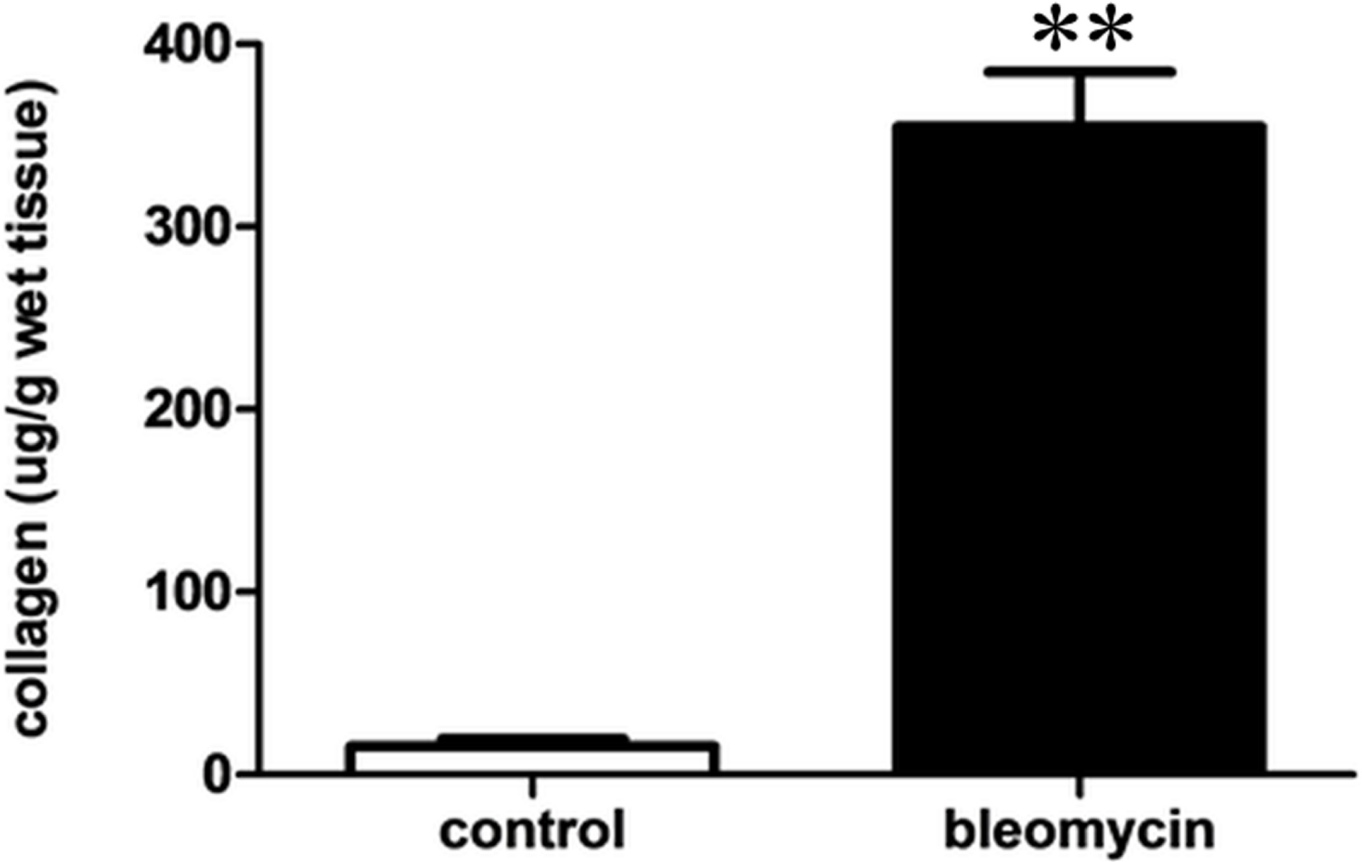
Collagen deposition was assessed using a SIRCOL assay of the lungs of bleomycin-induced fibrosis model and control model. Values are presented as mean ± SEM. Bleomycin vs. control (354.9 ± 90.3 vs. 15.4 ± 10.2 μg; **P < 0.001).

Collagen content was estimated using the SIRCOL collagen assay in lung tissues, as a means of quantifying the severity of fibrosis. The total collagen assay (μg/g tissue) showed a significant, above 10-fold, increase in the rats that received bleomycin, compared to the saline-treated control group (354.9 ± 90.3 vs. 15.4 ± 10.2 μg for bleomycin group vs. control group; *P* < 0.001; Fig 2).

### TLR expression in pulmonary sensory neurons in control and bleomycin model

**Fig 3:**
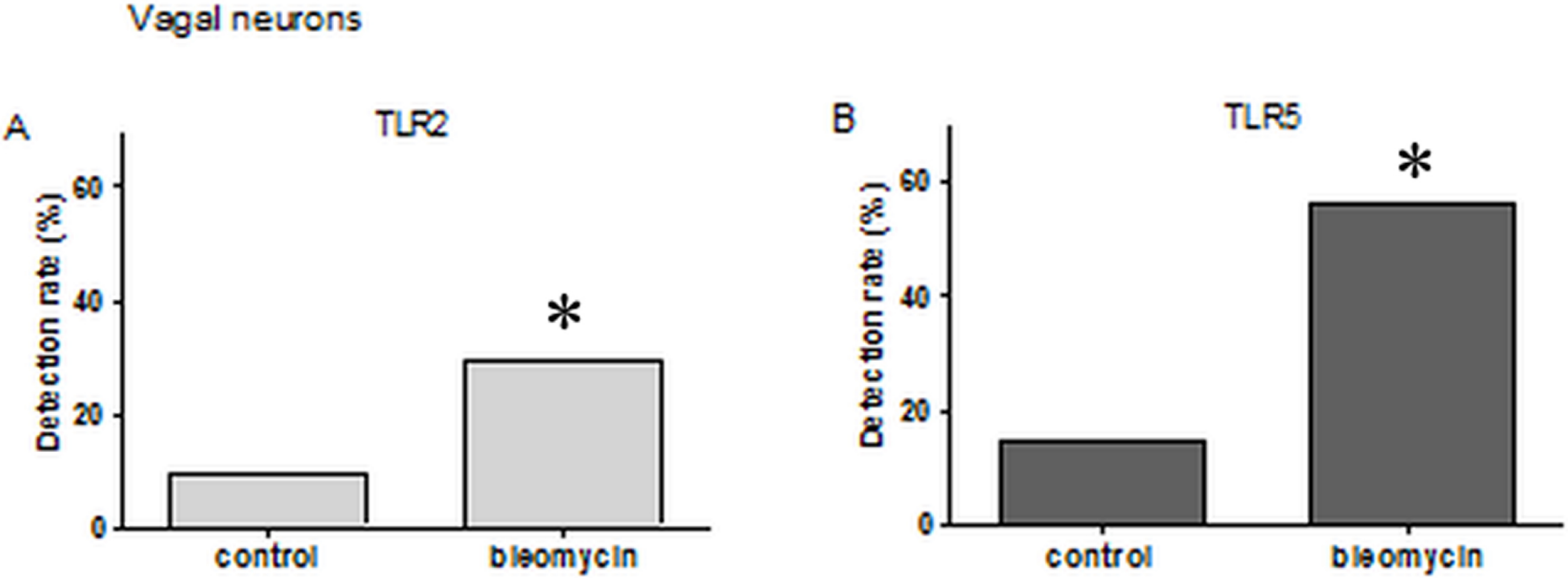
Single cell reverse transcription polymerase chain reaction of labeled nodose/jugular neurons for detection of toll-like receptor (TLR). **A**. The percentage of the TLR2 expressing neurons (29.5%) was significantly higher in the bleomycin-induced fibrosis model compared to the control group (9%; *P < 0.05; χ^2^-test). **B**. The percentage of the TLR5 expressing neurons (55.7%) was significantly higher in the bleomycin-induced fibrosis model compared to the control group (15.1%; *P < 0.05; χ^2^-test).

**Fig 4:**
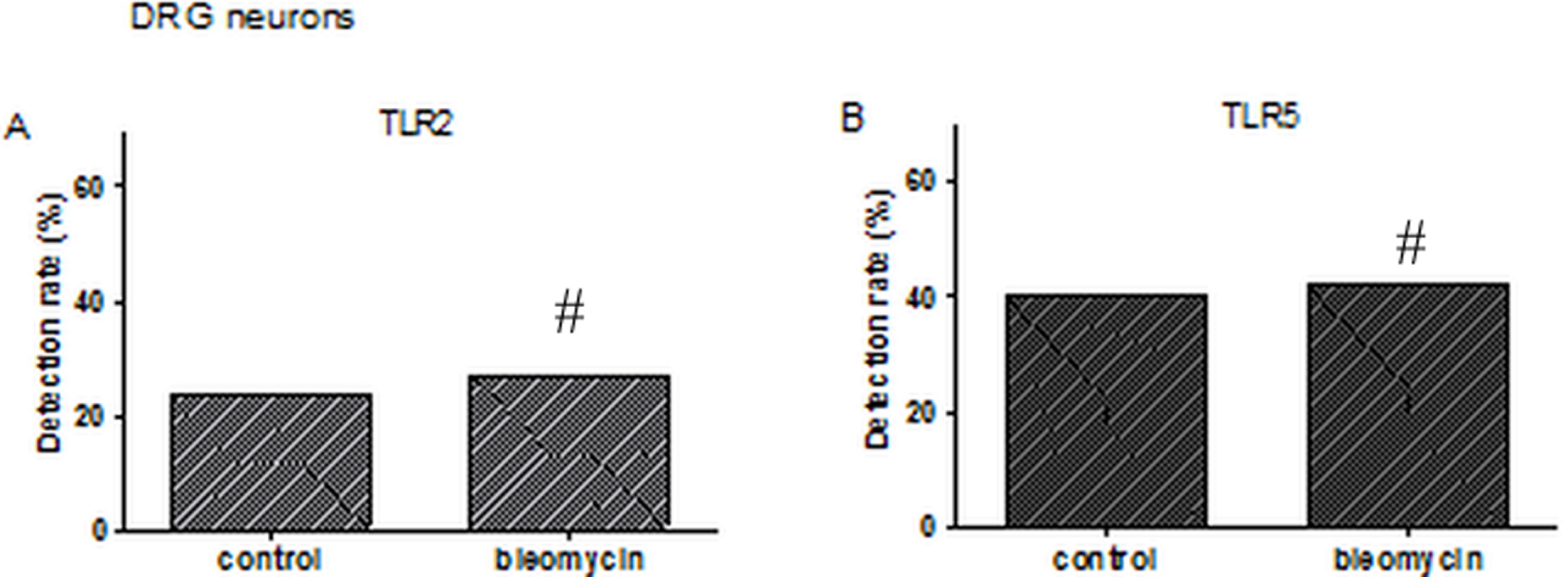
Single cell reverse transcription polymerase chain reaction of labeled dorsal root ganglion (DRG) neurons for detection of toll-like receptor (TLR). **A**. There was no significant difference between the percentage of TLR2 expressing neurons between the bleomycin-induced fibrosis model group and the control group (26.9% vs. 23.3%, ^#^*P* > 0.2; χ^2^-test). **B**. There was no significant difference between the percentage of TLR5 expressing neurons between the bleomycin-induced fibrosis model group and the control group (42.3% vs. 40.0%, ^#^*P* > 0.2; χ^2^-test).

DiI labeled neurons were collected from different rats. Single cells were collected using a micropipette and subjected to single cell RT-PCR. This analysis showed that, of all varieties of TLRs (TLR1-9), pulmonary sensory neurons expressed only TLR2 and TLR5. TLR2 transcripts were detected in 29.5% (18/61) and 26.9% (21/78) of DiI labeled pulmonary nodose/jugular neurons and DRG neurons in bleomycin-induced fibrosis model, respectively. TLR5 was also detected in 55.7% (34/61) and 42.3% (33/78) of pulmonary nodose/jugular neurons and DRG neurons of bleomycin-induced model, respectively. However, Other TLRs (TLR1–9, except TLR2 and TLR5) were barely expressed in pulmonary sensory neurons. In the saline-treated control group, TLR2 was expressed in 9% (3/33) and 23.3% (7/30) of the above neurons, respectively. TLR5 was expressed in 15.1% (5/33) and 40% (12/30) of these cell types, respectively. The expression level of TLR2 and TLR5 was significantly higher in nodose/jugular neurons of the bleomycin-induced model compared to those of the control model (*P* < 0.05 in bleomycin vs. control, Fig 3, S1 File). However, there was no significant difference between TLR2 and TLR5 expression in DRG neurons between groups (*P* > 0.2, in bleomycin vs. control, Fig 4, S1 File).

### TRP expression and co-expression of TRP and TLR in pulmonary sensory neurons in the bleomycin model

**Fig 5:**
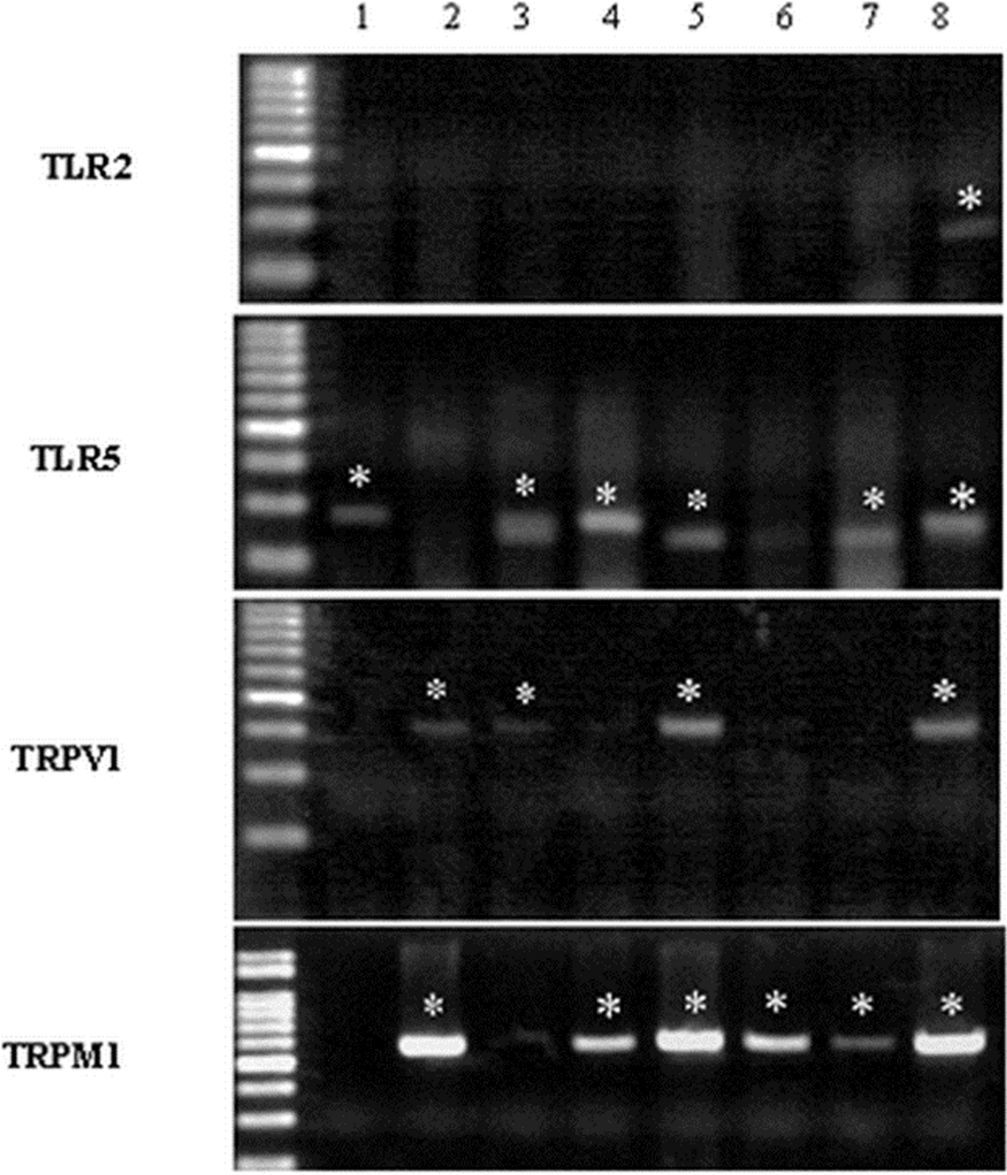
Identification of pulmonary sensory neurons using labeling of the rat ganglia and detection by single-cell reverse transcription polymerase chain reaction of the expression of toll-like receptors (TLRs) and transient receptor potential (TRP) channels in traced afferent neurons. Individual DiI-labeled neurons were collected in single PCR tubes. RT-PCR was carried out to detect the presence of TLR2, TLR5, TRPV1, and TRPM8. Results are representatives of those obtained in experiments. Asterisk indicates TLR and TRPV expressing neurons.

In the bleomycin-induced rat model, single cell RT-PCR showed expression of TLR2, TLR5 and TRP channels in pulmonary sensory neurons (Fig 5). Transient receptor potential ankyrin 1 (TRPA1), transient receptor potential vanilloid 1 (TRPV1) and transient receptor potential melastatin 8 (TRPM8) were detected in 19.5% (8/41), 38% (19/50) and 14.6% (6/41) of nodose/jugular neurons, respectively. In the same model, TRPA1, TRPV1 and TRPM8 were detected in 26.3% (10/38), 47.8% (22/46) and 42.5% (17/40) of DRG neurons, respectively. 50% (3/6) of TRPM8 positive neurons expressed TLR5, 68.4% (13/19) of TRPV1 neurons expressed TLR5 and 62.5% (5/8) of TRPA1 expressed TLR5 in nodose/jugular ganglion in bleomycin induced fibrosis model. In DRG neurons, 41.2% (7/17) of TRPM8 positive neurons expressed TLR5, 59.1% (13/22) of TRPV1 neurons expressed TLR5 and 44.4% (4/9) of TRPA1 neurons expressed TLR5 in the bleomycin-induced fibrosis model. With regard to TLR2, co-expression with TRP channel was relatively low. In the bleomycin-induced fibrosis model, 16.6% (1/6) of TRPM8 positive neurons expressed TLR2, 31.5% (6/19) of TRPV1 neurons expressed TLR2, and 12.5% (1/8) of TRPA1 expressed TLR2 in nodose/jugular ganglion. In the bleomycin-induced fibrosis model, 23.5% (4/17) of TRPM8 positive neurons expressed TLR2, 18.2% (4/22) of TRPV1 neurons expressed TLR2, and 44.4% (4/9) of TRPA1 neurons expressed TLR2 in the DRG (S1 File).

## Discussion

Airway sensory nerves are known to express several receptors and channels that are activated by exogenous and endogenous mediators causing coughing. Identification of the receptors and ion channels that are involved in coughing may lead to clinical implications. Although abundant studies investigating a variety of receptors and channels, including the TRP channel, were conducted, there has been no study investigating TLR expression in pulmonary sensory neurons. This study observed that pulmonary sensory neurons express the TLR varieties, TLR2 and TLR5. The expression of TLR2 and TLR5 was enhanced especially in vagal neurons in the bleomycin-induced fibrosis model group compared to the saline-treated control group. We also found evidence of co-expression of TLR5 and TRP channels in pulmonary sensory neurons. To our knowledge, this is the first report of TLR expression in any pulmonary sensory nerves.

TLRs are typically expressed in immune cells where they mediate innate immunity through recognition of pathogen-associated molecules [6, 7]. Most TLRs require the intracellular adaptor protein myeloid differentiation primary response gene 88 (MyD88) to mediate downstream signaling via activation of NF-kB and MAP kinase pathways, leading to the synthesis of inflammatory mediators including cytokine [22]. TLR5 has recently been proposed to play a role in the immunopathogenesis of asthma. Bacterial flagellin protein can act as a TLR5 agonist. TLR5 activation induces early and protective innate immune responses against microbes during lung infection. It was recently reported that flagellin and TLR5-activation in the airways can promote allergic asthma by priming allergic sensitization to allergens [23]. In a previous study, TLR5 was identified in multiple cells in the human airway, such as bronchial epithelium, alveolar type II pneumocytes, plasma cells, macrophages and neutrophils but not in airway neurons [24]. In our study, however, TLR5 was found to be expressed in pulmonary sensory neurons. This might suggest that the TLR5 expressed in vagal and DRG neuron is dominantly innervated by the lung rather than the airway. The bleomycin-induced fibrosis model is a representative model of IPF, which is not an airway disease like asthma. Hence, this novel observation, that TLR5 expression increases in pulmonary sensory neurons in the bleomycin-induced fibrosis model, suggests that there are different pathogenetic mechanism and clinical consequences from TLR5 function in asthma.

In addition to playing a role in the immune reaction and general inflammation, TLRs also play an important role in neuroinflammation. Neuroinflammation has been implicated in the development and maintenance of chronic pain and itch. This role in neuroinflammation is thought to involve the activation of TLRs in immune cells, glial cells and sensory neurons. The expression and function of several TLRs in neuronal cells has recently been demonstrated [9, 10, 21, 25]. TLR2 and TLR4 have been shown to modulate spinal cord glial activation and contribute to the development of neuropathic pain following nerve injury and chemotherapy [8, 26]. TLR3, TLR4 and TLR7 were also found in primary sensory neurons and these receptors play a role in detecting exogenous pathogens or endogenous danger signals that can induce pain and itch [21, 27]. In this study, the expression of all TLRs in sensory neurons innervating the lung were examined. Interestingly, this study showed an increase in TLR2 and TLR5 expression in pulmonary nodose/jugular neurons and DRG neurons in both the control group and the bleomycin-induced fibrosis group. There are a few studies investigating TLR2 and TLR5 in terms of pain and itch. One study reported that TLR5 mediated A-fiber blockade inhibiting mechanical allodynia in neuropathic pain [9]. Chronic pain is a hypersensitivity state that results from peripheral and central sensitization. Several reports have suggested that chronic itch and chronic cough are also similar hypersensitivity states [28, 29]. Neuroinflammation has been implicated in the development and maintenance of chronic pain, itch, and cough, through the activation of TLRs in immune cells, glial cells and sensory neurons [11]. Inferring that a similar mechanism applies here, the expression of TLR2 and TLR5 in pulmonary sensory neurons may be considered to be associated with cough.

This study also showed that TLR2 and TLR5 expression in nodose/jugular neurons was significantly increased in the bleomycin-induced fibrosis model group compared to the saline-treated control group. It was interesting to observe that no enhanced expression of TLR was found in DRG neurons in the bleomycin-treated group compared to the control group. The neurons in the DRG innervate various types of organs and tissues and, especially thoracic and lumbar DRG, contain the primary sensory neurons innervating the thoracic and abdominal visceral organs, including the lungs [30]. However, it is known that the nodose and the jugular ganglia of the vagal nerve provides a major source of lung sensory innervation [1, 30, 31]. Several previous studies have reported that activated vagal sensory neurons may induce neurogenic inflammation and an activated system lead to the up regulation of cough response [11, 32]. This result suggests that the expression of TLR2 and TLR5 on vagal nerves has the potential to be enhanced in certain pathophysiological conditions and that the level of expression might play a functional role in pathologic lung disease and clinical symptoms.

Members of the TRP family of ion channels are present on vagal sensory nerves, which, when activated, initiate the cough reflex. TRP channels are a family of ion channels which act as cellular sensors and respond to changes in the external environment. TRP channels are known as cellular sensors because they respond to environmental changes including temperature, stretch, chemicals, oxidation, osmolarity and pH [2]. Members of the TRP family of ion channels have emerged as strong candidates for sensory receptors that modulate the cough reflex. TRPV1, TRPA1 and TRPM8 have been shown to be functionally expressed in vagal ganglia [33–35]. Consistent with the previous report, single cell RT-PCR showed that TRPA1, TRPV1, and TRPM8 are expressed in pulmonary vagal neurons and DRG neurons.

This study also demonstrated that some pulmonary neurons co-express both TLR5 and TRPV1 and TRPM8 and TRPA1. In previous reports, TRP channels, which mediate neurogenic inflammation in sensory neurons, have been identified as being expressed and functional in non-neuronal cells, such as airway epithelial cells [36, 37]. Co-expressed TRPV1 and TRPM8 have been localized to nerve endings in human airways. The expression of TRPV1 and TRPM8 is increased in patients with chronic cough [38–40]. Several of the reviews that have been mentioned share similarities in chronic pain, itch and cough. Stimulation of these TLRs in sensory neurons mediates pain and itch, and one study identified the co-expression and interaction of TLR and TRPV1[27, 41–43]. Activation of TLR4 using LPS in DRG neurons does not trigger inward currents or action potentials, although TLR4 modulates a histamine-induced itch through transcriptional control of TRPV1 expression [44]. Tissue injury results in the release of miRNAs, such as let-7b, from damaged and inflamed tissue. Let-7b binds to TLR7 (step-1), which is coupled with TRPA1 (step-2) on the surface of nociceptive neurons. Activation of TLR7 and TRPA1 by let-7b increases the excitability of sensory neurons (step-3), leading to pain and peripheral sensitization [45]. Considering that the mechanism of pain, itch and cough are similar, the results of this study raise the possibility that there could be an interaction between TLR5 and the TRP channel in pulmonary neurons.

This study has taken a de novo approach and has shown very influencing results in spite of several limitations. Limited samples may have affected the accuracy of our results. And the presence of mRNA transcripts does not necessarily reflect the abundance or even presence of the associated protein, although RT-PCR has high sensitivity. Future studies, including immunochemistry and application of material like TLR agonists, are warranted to clarify whether increased TLR2 and TLR5 expression implies an increase in TLR function, if the expression of the downstream genes of TLR2 and TLR5 signaling is altered and if neuronal TLR expression is directly associated with respiratory symptoms like cough.

In conclusion, this study provides evidence that TLR2 and TLR5 are present in pulmonary sensory neurons and that TLR2 and TLR5 expression is enhanced in vagal neurons in the bleomycin-induced fibrosis model. This study also reported the co-expression of TLR5 and TRP channels in pulmonary sensory neurons. This co-expression might be involved in controlling and manifesting the clinical symptoms, such as cough. To understand the role of TLRs in pulmonary sensor nerves, further study will be required.

## Supporting information

S1 File Supplemental information. (XLSX)
